# Evaluating User Compliance in Mobile Health Apps: Insights from a 90-Day Study Using a Digital Sleep Diary

**DOI:** 10.3390/diagnostics13182883

**Published:** 2023-09-08

**Authors:** Hlín Kristbergsdottir, Lisa Schmitz, Erna Sif Arnardottir, Anna Sigridur Islind

**Affiliations:** 1 Department of Psychology, Reykjavik University, 102 Reykjavík, Iceland; 2 Reykjavik University Sleep Institute, 102 Reykjavík, Iceland; ernasifa@ru.is (E.S.A.); annasi@ru.is (A.S.I.); 3 Department of Computer Science, Reykjavik University, 102 Reykjavík, Iceland

**Keywords:** mobile sleep diary, digital health, sleep revolution, telemedicine, apnea–hypopnea index, compliance

## Abstract

Sleep diaries are the gold standard for subjective assessment of sleep variables in clinical practice. Digitization of sleep diaries is needed, as paper versions are prone to human error, memory bias, and difficulties monitoring compliance. Methods: 45 healthy eligible participants (M^age^ = 50.3 years, range 23–74, 56% female) were asked to use a sleep diary mobile app for 90 consecutive days. Univariate and bivariate analysis was used for group comparison and linear regression for analyzing reporting trends and compliance over time. Results: Overall compliance was high in the first two study months but tended to decrease over time (*p* < 0.001). Morning and evening diary entries were highly correlated (r = 0.932, *p* < 0.001) and participants significantly answered on average 4.1 days (95% CI [1.7, 6.6]) more often in the morning (M = 60.2, sd = 22.1) than evening ((M = 56.1, sd = 22.2), *p* < 0.001). Conclusion: Using a daily diary assessment in a longitudinal sleep study with a sleep diary delivered through a mobile application was feasible, and compliance in this study was satisfactory.

## 1. Introduction

Sleep is one of the fundamental pillars of good health. It impacts a range of physiological functions, such as the hormonal system, cardiovascular system, immune system and brain health [1]. Sleep disorders cause a major socioeconomic burden on healthcare systems worldwide, e.g., using the current diagnostic criteria, almost 1 billion people between the ages of 30 and 69 are affected by obstructive sleep apnea (OSA), the most common type of sleep-disordered breathing [2]. This manifests in billions of USD annually in lost productivity, cost of accidents, and other downstream health sequelae [3]. There is profound evidence that sleep disorders are associated with a range of physical health problems, such as diabetes, cardiovascular disease, obesity, and hypertension, that lead to increased mortality and morbidity rates [2,4,5,6]. Individuals with untreated sleep disorders are also at higher risk of deteriorating cognitive functioning that may impact occupational performance and social participation, thus compromising the quality of life and individuals’ socioeconomic status [7,8,9]. Sleep disorders are strongly associated with cognitive decline, attention loss, memory impairment, issues with mood regulation, and mood disorders [8,10,11]. Therefore, sleep disorders are one of the leading health challenges today and a serious threat to public health if left undiagnosed and untreated.

Our understanding of sleep disorders and possible underlying mechanisms is challenging. We still face several limitations in sleep research, i.e., expensive and time-consuming diagnostic procedures, small and homogeneous sample sizes, lack of long-term studies on sleep disorders, and daily fluctuations in sleep [7,12,13]. Hence, while the impact of short-term and acute sleep deprivation has been well-documented, our understanding of long-term sleep disorders with daily fluctuations in a naturalistic setting (i.e., home environment) needs to be clarified [7,14,15].

Although polysomnography (PSG) is considered the gold standard for sleep measurements, it is time-consuming and expensive. Moreover, it does not include subjective feedback from the patient, which can be pivotal for aspects like sleep hygiene and insomnia diagnosis. Additionally, PSGs require manual scoring, and the queues for studies tend to be lengthy and the overall resources are scarce. Continuous measurement for long-term studies of sleep in a naturalistic setting would be revolutionary [16]. Furthermore, a combination of subjective and objective measurements would be beneficial as both provide their own unique sleep features [17,18]. When it comes to subjective sleep measurements, sleep diaries and self-report measurements are more compatible with long-term and continuous measurements, where the long-term effect and compliance over time are studied [16]. Sleep questionnaires and sleep diaries outline the gold standard for subjective self-reported assessment of sleep variables in clinical practice. Sleep diaries are traditionally delivered on paper and have shown a high correlation with actigraphy measures on sleep onset and offset times, making them an essential support tool for diagnosing and treating sleep hygiene and insomnia [19,20]. However, although sleep diaries in pen-and-paper format are the conventional method for self-report sleep assessment, they are increasingly considered outdated and are prone to memory bias and difficulties in monitoring compliance [6,21,22,23]. Despite the rapid increase in digitization across different sectors, the consumer adoption of mobile apps and wearables, and their feasibility for delivering digital health solutions, there remain significant challenges in the design and development of digital health solutions for sleep, in particular in digital sleep diaries [16,24].

The adoption of digital health solutions and the rapid change towards more personalized and participatory healthcare is promising [25,26]. It enables more intensive and larger data collection than is possible with traditional data collection in sleep research. However, digitization of healthcare in general and for sleep, in particular, has been lagging significantly [7], although it is evident that digital health solutions outline an important factor for the future [26]. With the development of digital health solutions, there is great potential for longitudinal subjective assessment of sleep health, particularly the ability to monitor in real time and follow up continuously with subjects between more extensive examinations. Healthcare providers and researchers can be updated about key indicators of clinically significant changes such as the subjects’ sleep and lifestyle habits, cognitive functioning, mood, and physical activity [25,27]. The benefits of digital health solutions include that they are cost-beneficial, improve accessibility, enable at-home monitoring for both clinical populations and the general population, and may provide new information in clinical evaluation and diagnostics that are currently not being captured [7,13,25,28]. For example, it is possible to measure symptoms more frequently and for longer periods by using digital health solutions for momentary assessments and, thus, to capture new domains of sleep that traditional in-laboratory assessments do not. A fundamental shift towards this new horizon of digital sleep health is imperative.

A mobile sleep diary has several benefits, including detailed real-time data on night-to-night variability in sleep and sleep habits [16,29]. A mobile sleep diary offers the possibility of reporting daily sleep measures close to the reported event, increasing data accuracy while minimizing the risk of recall bias [30]. Digital sleep diaries enable both patients and healthcare providers to evaluate their sleep measures and follow their progress in real time through informative data visualizations. However, the main concern and possible limitation of using sleep diaries (either on paper or digital), especially when used for a long consecutive period, is compliance [16,30]. Few studies have examined the usage of a digital sleep diary for prolonged periods (>7 nights) [13,31,32], and we found only one study, Thurman et al. [13], that investigated compliance specifically over a 16-week period. In that study, compliance decreased linearly over time.

The primary aim of this study was to examine the feasibility and provide a data-driven analysis of a longitudinal repeated mobile sleep diary assessment. Our mobile app includes a sleep diary which was co-designed and developed as a part of the Sleep Revolution mobile application (app), based on the extended paper-and-pen Consensus Sleep Diary [21,24]. We examined the compliance in terms of reporting in the app for 90 consecutive days without any active intervention for a group of healthy sleepers and subjects with reported sleep problems. We hypothesized that compliance would decrease significantly over time according to a linear function. As a secondary exploratory aim, we examined individual differences in compliance across different types of sleep measures and demographic groups. We hypothesized that individuals with sleep apnea or insomnia were more likely to show higher levels of compliance and that compliance would not vary among different demographic groups. As a third exploratory aim, we examined if compliance would differ between weekdays and weekends, i.e., we hypothesized that individuals would be less compliant during weekends than on weekdays.

## 2. Materials and Methods

### 2.1. Study Participants

Fifty-nine participants were recruited by word of mouth and local advertisement in Iceland (within Reykjavik University, newspapers, radio, etc.) for this three-month pilot study. Inclusion criteria were that participants had to be of age 18 or older, own a smartphone and be willing to download the Sleep Revolution app, and not shift workers. Participants were asked to (i) complete an online questionnaire on background information and self-report sleep and lifestyle measures, (ii) complete a three-night self-applied polysomnography, and (iii) complete a sleep diary twice a day. A total of 45 participants (mean age = 50.3 years, range 23–74, 25 females, 20 Males) completed all three tasks and were included in the analysis. Those not included dropped out of the study at the beginning and did not complete the questionnaires or the three-night self-applied polysomnography. Participants were not paid or compensated in any form for their study participation.

### 2.2. Design and Procedure

Prior to participation in the main sleep study, participants completed questionnaires on demographics, lifestyle, and self-reported sleep measures as well as screening lists. Participants were then subjected to a three-night self-applied polysomnography and asked to keep a daily sleep diary in the Sleep Revolution app. Automated reminders were sent to participants to enhance compliance on a regular basis. Participants were only allowed to answer on the current day to ensure they reported their real-time experience. Participants signed an informed consent prior to their participation and the study was approved by the Icelandic National Bioethics Committee (ref. no. 21-070).

### 2.3. Measures

#### 2.3.1. Sleep Diary

The app was co-designed and developed through extensive user engagement within the Sleep Revolution project. The co-design approach outlines a novel way of approaching the design, as we have elicited the needs of the participants iteratively throughout the design process [33,34]. The design matured through multiple iterations and eventually, the app was ready for inclusion of participants. The results of this study outline the first cohort using the app in a longitudinal manner. In this study, the participants were instructed to complete the sleep diary twice a day for 90 consecutive days in an app developed by the Sleep Revolution [7,24]. The sleep diary in the mobile app is adapted from the Consensus Sleep Diary [21] and collects a subjective evaluation of sleep measures. In addition, the Sleep Revolution sleep diary also gathers information on lifestyle factors that might impact their sleep, i.e., mood and stress levels, number of naps, number of caffeine and alcohol units consumed, and duration of exercise conducted that day.

#### 2.3.2. Three-Night Self-Applied Polysomnography

A self-applied polysomnography [35,36,37] was used at baseline over three consecutive nights and the apnea–hypopnea index (AHI) was derived. AHI was defined as the number of respiratory events (apneas or hypopneas) per hour of sleep. For adult subjects, AHI is traditionally classified as normal (AHI < 5/h), mild (AHI between 5 and 14.9 events per hour), moderate (AHI between 15 and 29.9/h), and severe (AHI ≥ 30/h). Due to this study’s relatively small sample size, an AHI score of ≥15 was considered the threshold criteria for having OSA for covariate analysis [14].

The recordings were annotated by expert sleep technologists in accordance with the AASM manual, version 2.6 [38]. To do this, the Noxturnal version 6.2.2 software (Nox Medical, Reykjavik, Iceland) was used. The N3 scoring rules were updated for the self-applied setup in accordance with Kainulainen et al. [36] and the recommended AASM rules for hypopnea scoring were used.

#### 2.3.3. Insomnia Severity Index (ISI)

The ISI is a 7-item self-report instrument designed to assess the severity of both nighttime and daytime components of insomnia. The recall period typically consists of the most recent month. The tool evaluates the severity of sleep onset, sleep maintenance, and early morning awakening problems, sleep dissatisfaction, interference of sleep difficulties with daytime functioning, noticeability of sleep problems by others, and distress caused by the sleep difficulties. Each of these items is rated on a 5-point Likert scale (e.g., 0 = no problem; 4 = very severe problem), with a total score ranging from 0 to 28. The interpretation for the total score is as follows: absence of insomnia (0–7); sub-threshold insomnia (8–14); moderate insomnia (15–21); and severe insomnia (22–28). In this study, a cut-off of ≥15 was used to indicate a significant risk of insomnia. The ISI is available in three versions—a version each for patients, clinicians, and significant others—but this paper focuses on the patient version only [39].

#### 2.3.4. Epworth Sleepiness Scale (ESS)

The ESS is an 8-item self-report instrument that assesses the likelihood of dozing or falling asleep in common situations of daily living. A total score is calculated and ranges from 0 to 24, where higher scores indicate a higher likelihood of dozing or falling asleep and scores of >10 indicate a significant risk of excessive sleepiness [40].

#### 2.3.5. Depression Anxiety and Stress Scale 21 (DASS-21)

The DASS-21 is a 21-item self-report instrument designed to measure the negative emotional states of depression, anxiety, and stress. Each subscale includes 14 items with the following cut-off scores as positive indicators for symptoms of depression (≥10), anxiety (≥8), and stress (≥15) [41].

#### 2.3.6. Covariates

Based on previous research, the following covariates that may relate to sleep and compliance were included in the analysis: age (years), sex (female/male), education (high/low), employment status (working/not working), weekly exercise (count), marital status of married or cohabitating (yes/no).

### 2.4. Statistical Analytics

Statistics were calculated using IBM SPSS Statistics 28 and Python. Background data are presented descriptively, categorical data by frequencies (n) and proportions (%) and continuous data with means and standard deviations (S.D.s). The perceived total sleep time refers to the answer to the question “In total, how long did you sleep?”. Participants had the option to have this value automatically calculated from the answers to the questions “What time did you try to go to sleep? ”, “How long did it take you to fall asleep?”, “In total, how long did these awakenings last?”, and “What time was your final awakening?”. The result of the calculated value is referred to as the estimated total sleep time. To compare the mean count of morning and evening diary entries, a paired sample T-test was applied and multiple linear regression was used to examine individual differences in morning and evening diary entries by demographics, sleep measures, and relevant lifestyle factors. To account for multiple comparisons, the Bonferroni correction test was used and the significance level was adjusted (*p*-value of 0.05/2 = 0.025). Simple linear regression was used to explore linear trends in morning and evening diary entries over time. The resulting linear regression estimate (ß) can be interpreted as the average percentage point change in compliance per day.

## 3. Results

### 3.1. Descriptive Statistics

Table 1 shows the descriptive sociodemographic profile of participants in the study.

### 3.2. Overall Compliance

Overall compliance was high over the study period. Participants answered a total of 2711 times out of the 4049 possible morning diary entries (67%) and 2525 times out of the 4050 possible evening diary entries (62%). Simple linear regression revealed a significant linear trend of compliance tending to decrease over time for both mornings (b = −0.675, t = −8.581, *p* < 0.001, R2 = 0.456) and evenings (b = −0.745, t = −10.464, *p* < 0.001, R2 = 0.554).

**Figure 1:**
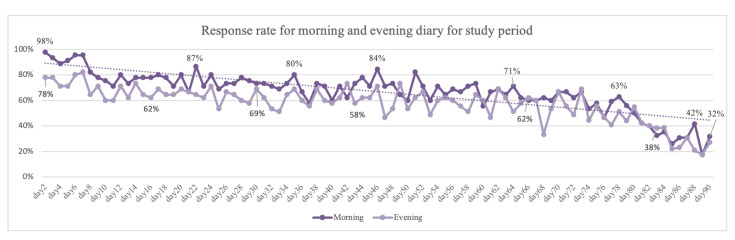
Daily compliance for morning and evening diaries for the study period.

**Figure 2:**
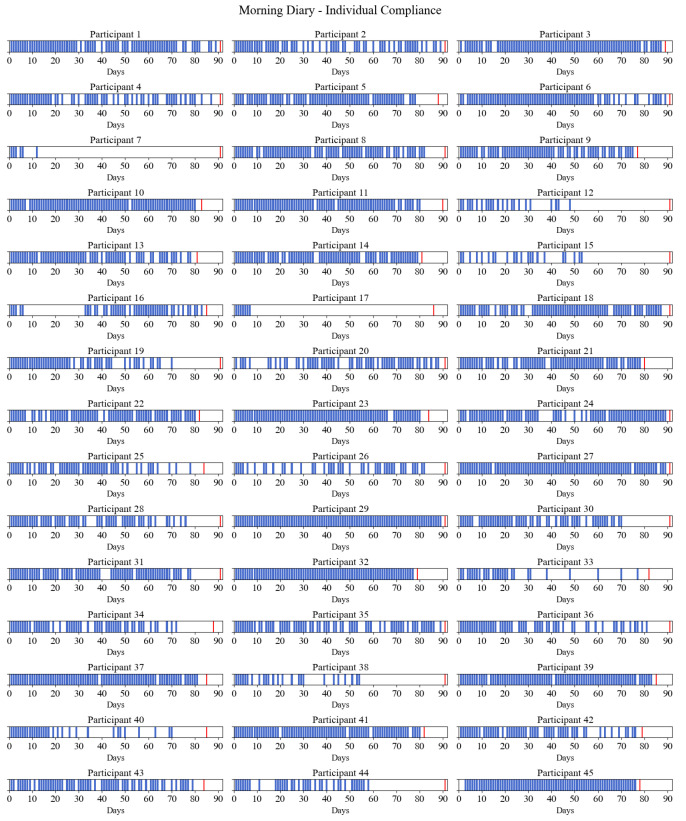
Visual presentation of individual compliance for the morning sleep diary. The blue bar marks the days the participants filled out the sleep diary. The red bar marks the end of the individual participant’s study period.

**Figure 3:**
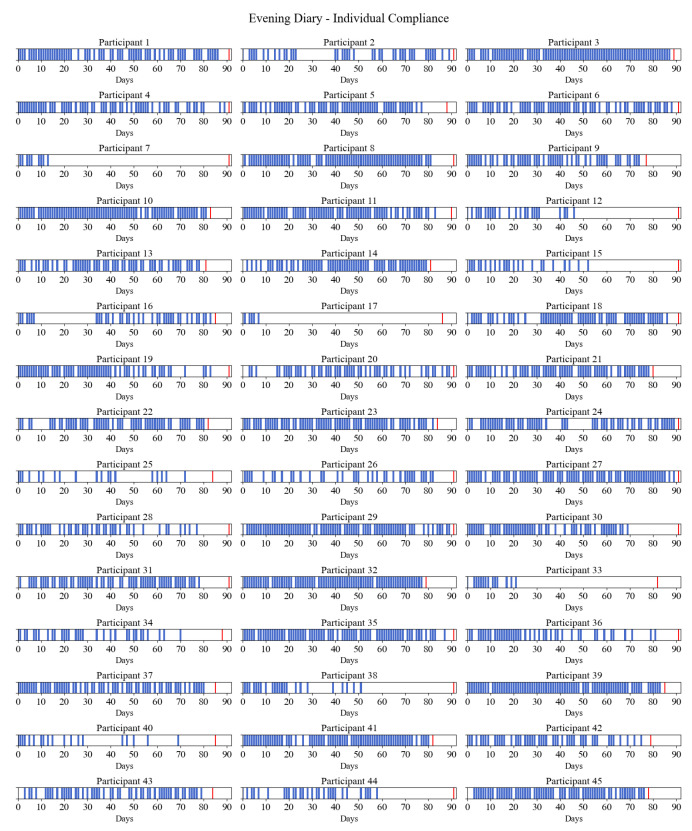
Visual presentation of individual compliance for the evening sleep diary. The blue bar marks the days the participants filled out the sleep diary. The red bar marks the end of the individual participant’s study period.

Compliance was high at the beginning of the study and stayed considerably stable through the first two months of the study. At the beginning of the study, compliance was 98% for the morning diary and 78% for the evening diary. In the first month, average compliance was high, at 80% (morning) and 67% (evening), and then slightly dropped between the first and second months, to 70% (morning) and 60% (evening). During the last month, compliance started to decline and dropped to 31% (morning) and 26% (evening) in the last days of the study (see Figure 1). Figure 2 and Figure 3 provide a visual presentation of compliance by each participant for the morning (see Figure 2) and evening (see Figure 3) sleep diaries.

Morning and evening diary entries were highly correlated (r = 0.93, *p* < 0.001), and results from paired sample *t*-test show a significant average difference (t = 3.38, df = 44, *p* < 0.002). On average, participants answered 4.1 (95% CI [1.7, 6.6]) days more often in the morning (M = 60.2, sd = 22.1) than in the evening (M = 56.1, sd = 22.2).

#### 3.2.1. Compliance by Individual Differences

We examined compliance for the morning and evening diaries by individual differences with simple linear regression, i.e., demographic profile, sleep measures, and mental health. For demographics, objective and subjective sleep measures, and symptoms of depression, anxiety, and stress, a significant mean difference in compliance was not found (see Table 2).

#### 3.2.2. Compliance on Weekdays and Weekend Days

We compared the overall compliance for weekdays and weekend days for the morning and evening diaries. Table 3 shows the average compliance rate for all participants during the study period consisting of 2892 weekdays and 1158 weekend days. The compliance rates show no significant difference between weekdays and weekends for the morning and evening diaries.

### 3.3. Total Sleep Time

The morning diary collects various data points regarding the participants’ sleep the previous night. We evaluated the gathered data and found discrepancies regarding bedtimes and the corresponding total sleep time. The implementation of the application allowed participants to enter what time they went to sleep and when they woke up in an am/pm format. A button to estimate the total sleep time based on the previously entered data was provided to the participants in the app view for entering the total sleep time.

We examined the deviation of the participants’ perceived total sleep time deviation from the estimated total sleep time. The data distribution is symmetrical around the mean, with most perceived total sleep time entries showing no deviation from the estimated total sleep time. However, a smaller second bell curve can be found around the −12 h deviation mark. A possible explanation for this observation could be that incorrect time entries cause this deviation due to the am/pm format they were entered in.

**Figure 4:**
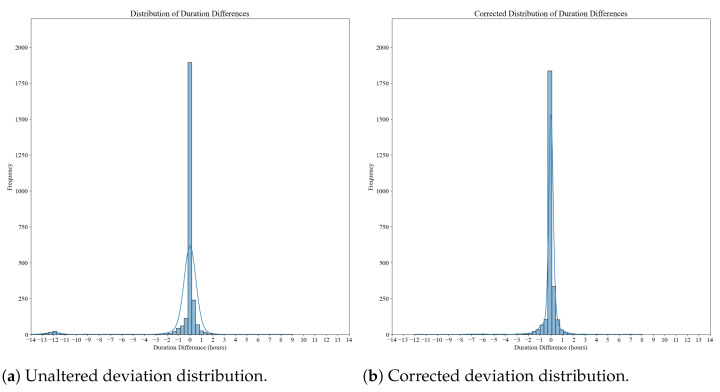
Histograms of the unaltered and corrected deviation distributions of the perceived total sleep time from the estimated total sleep time.

Therefore, we also examined the distribution after correcting for the outliers. We corrected the data by adding 12 h to those deviation data points that show a negative correction greater than 8 h and subtracting 12 h from those that show a positive correction greater than 8 h. This eliminates the majority of outliers and results in a bell curve with fewer outliers, as can be seen in Figure 4.

The data (see Figure 4) show that over 90% of the total sleep time entries did not deviate more than an hour from the estimated duration.

## 4. Discussion

The current study examined the feasibility of using a sleep diary in an app for 90 consecutive days and individual differences in sustaining compliance. To our knowledge, a study of this magnitude has not been performed previously, and our findings provide a novel addition to the existing literature on digital measures in sleep research.

Our main findings showed high compliance rates, especially for the first two months of the study (98–62%), and that the digital sleep diary was accessible to all, regardless of demographic profile, possible sleep problems, or symptoms of depression, anxiety, and stress. However, compliance did significantly decline over time, as expected. At the beginning of the study, approximately 98% of study participants completed their morning sleep diary entries, and 78% completed their evening diary entries. By the end of the study, 52–46% of participants remained compliant. For the last days of the study, compliance rates dropped to 26–31%, indicating participants experienced a higher respondent burden. The decrease in compliance could be attributed to participants’ fatigue or a lack of motivation to continue reporting over an extended period. Another explanation could be that participants were asked to attend a final interview at the end of the study and were able to choose a date that fit them well. Some participants chose a date before they had finished their 90 days and likely stopped using the app after the interview. This is a limitation to the study and the interpretation of compliance, as only 50% of the total sample used the app for the 90-day study period. As there was no special endpoint or treatment being monitored in the study, this could have affected compliance. Nevertheless, we find the results interesting and novel despite this limitation as, to our knowledge, findings on compliance in a digital sleep diary for a three-month study period have not been reported.

Despite the immense knowledge of short-term and acute sleep problems and their effects on physical and mental health [2,42], previously, studies have not focused on daily fluctuations in sleep in a naturalistic setting for prolonged periods of time [13,15], e.g., in a recent systematic review of the relationship between daily sleep and mood, results show that the majority of studies lend support to a short-term dynamic association between sleep and daily mood, but studies of the long-term effects are few and still remain to be fully investigated [43].

Studies using a digital sleep diary have mostly reported findings on compliance for up to 14 days [30]. One study conducted by Thurman et al. [13] that examined compliance in using a digital sleep diary for 16 consecutive weeks showed compliance significantly decreased with time, consistent with our findings. While our sleep diary was designed with the goal of fostering engagement through notification reminders and data visualizations, additional measures should be taken to ensure high compliance for longer time periods. The app version that was used during the study period lacked actionable feedback, but another study in the Sleep Revolution is currently ongoing and investigates participant compliance and habit change through the use of goals as part of the gamification of the digital sleep diary. Studies have shown that the reliability of data collected by sleep diaries increases with the number of days used and is therefore important to support participants’ motivation in answering the sleep diary for prolonged periods [31,32,44].

Our findings also revealed a high correlation of compliance between morning and evening diary entries, indicating consistency in participants’ reporting. However, participants answered the morning diary more frequently than the evening diary, despite the presence of notification reminders in the app for both morning and evening diaries. This disparity may be attributed to the circumstance that the morning reminders were received at a more convenient time. It is conceivable that participants were more likely to be at home in the morning and, thus, had easier access to the diary, while in the evening, they might have been occupied by various activities. To our knowledge, such findings of a correlation between compliance in the morning and evening entries of a digital sleep diary have not been reported elsewhere.

Interestingly our findings also revealed that compliance on weekdays and weekends showed no significant differences for either morning or evening diaries, suggesting that participants maintained consistent reporting habits regardless of the day of the week. The expectation was that compliance would decrease during the weekend, as people’s schedules tend to be more irregular on those days than they are during weekdays. A possible explanation for the consistent compliance is the notification reminders that helped people to remember to fill in the diary during weekdays and weekends alike. Our findings suggest that a digital sleep diary supports data reliability. Previous studies have mainly focused on the accuracy of data collection between weekdays and weekends, suggesting a need for a minimum of 7–10 days of entries in order to obtain reliable data, especially on weekends [31,32]. However, findings on the difference in compliance during weekdays and weekends have not been reported elsewhere to our knowledge.

Lastly, our findings revealed discrepancies between participants’ perceived total sleep time and the estimated total sleep time. The distribution of the deviation showed a symmetrical curve, indicating that most participants accurately estimated their total sleep time. However, a smaller portion of participants showed significant deviations, likely due to errors in entering sleep and wake times or confusion related to the am/pm format, which is generally not used in Iceland. Correcting for these outliers resulted in a more normalized distribution, suggesting that providing clearer instructions or formats for time entry could improve accuracy in estimating total sleep time.

### Strengths and Limitations

The study has several strengths, including addressing gaps in the literature on digital solutions for sleep research. To our knowledge, this is the first study that investigates participant compliance with a sleep diary app in an intensive longitudinal setting. The results highlight the potential of digital health solutions in improving longitudinal sleep assessment and indicate that a digital sleep diary can foster compliance and, thus, overcome the limitations and drawbacks associated with the traditional pen-and-paper format [6,21,22,23]. The study emphasizes that the integration of longitudinal subjective measurements in digital health solutions opens up new possibilities for capturing comprehensive sleep information and contributing to meaningful changes in daily habits, mood, and overall well-being [8,10,11,16].

Furthermore, the study has addressed the lack of long-term studies in naturalistic settings [7,12,13,16] by evaluating digital solutions. By providing more accessible and cost-effective methods of sleep assessment, digital health solutions have the potential to mitigate the socioeconomic burden of sleep disorders on healthcare systems and improve overall public health outcomes [2,3,4,5].

Overall, the findings from this study provide valuable insights into compliance and sleep patterns among participants using a morning and evening sleep diary app. However, there are limitations to consider. First, the sample size in this study was relatively small, and our findings need to be interpreted with caution, as generalizability in such samples is limited. Future studies with larger and more diverse participant samples could provide a more comprehensive understanding of compliance and sleep duration patterns. Second, compliance in the study dramatically decreased in the last days of the study period, which could impact the reliability of the data. The fact that some participants attended a final interview before they had finished the study might explain the dramatic drop in compliance in the last 10 days of the study. In future studies within an intensive longitudinal setting, it might be beneficial to provide more frequent reminders and positive feedback to participants to promote their participation and promote the quality of data received. In addition, a final interview should occur only after participants have finished the study to ensure compliance throughout the study period. Lastly, the accuracy and quality of data collected in a sleep diary mobile application need further testing. In our study, a small portion of participants showed significant deviations when entering sleep and wake times. This is likely due to the format being am/pm, but further testing on the reliability of the data being collected overall in a mobile application would be beneficial.

## 5. Conclusions

By providing more accessible and cost-effective methods of sleep assessment, digital health solutions have the potential to mitigate the socioeconomic burden of sleep disorders on healthcare systems and improve overall public health outcomes [2,3,4,5]. Thus, the findings from this study provide valuable insights into compliance and sleep duration patterns among participants using a morning and evening sleep diary app.

In conclusion, this study has demonstrated the potential of digital sleep diaries in revolutionizing sleep assessment and management. The integration of subjective measurements in an app holds promise for enhancing diagnostic procedures, designing targeted interventions, and ultimately improving the quality of life for individuals affected by sleep disorders. Ergo, using daily diary assessment in a longitudinal sleep study with a sleep diary delivered through an app is feasible, and compliance in this study showed to be satisfactory. Moreover, this paper contributes a compliance assessment of the novel sleep diary delivered through a mobile application. The results show that the app holds high potential both for the future of data collection in sleep medicine and clinical assessment.

## Figures and Tables

**Table 1:** Measurements in morning and evening sleep diary app.

Variable	Total (N = 45) M ± SD ^1^/%
**Demographics and lifestyle**	
Age, M (SD)	50.3 ± 11.7
Sex, Female	55.6
Education, high	66.7
Work status, employed	66.7
Marital status, married/cohabitating	91.1
Weekly exercise, M (SD)	2.8 ± 2.00
**Sleep measures**	
AHI (≥15 events per hour) ^2^	26.2
ISI score (≥15) ^3^	31.1
ESS score (>10) ^4^	42.2
**Mental health (DASS-21 ^5^)**	
Depression score (≥10)	20.5
Anxiety score (≥8)	13.6
Stress score (≥15)	20.5

^1^ Mean and standard deviation, ^2^ Apnea–hypopnea index, ^3^ Insomnia Severity Index, ^4^ Epworth Sleepiness Scale ^5^ The Depression, Anxiety and Stress Scale—21 Items.

**Table 2:** Simple linear regression results for morning and evening diary entries.

	Morning Diary Entries						Evening Diary Entries					
Variables	R^2^	Un-Standardized Coefficients		Standardized Coefficients	t	* p *	R^2^	Un-Standardized Coefficients		Standardized Coefficients	t	* p *
		B	SE	Beta (*β*)				B	SE	Beta (*β*)		
**Demographics**												
Age	0.087	0.594	0.298	0.294	1.995	0.053	0.097	0.630	0.297	0.311	2.118	0.040
Sex, female	0.016	5.570	6.645	0.127	0.838	0.497	0.041	8.930	6.594	0.202	1.354	0.183
Marital status, cohabitating	0.000	1.640	11.695	0.021	5.263	0.889	0.000	1.220	11.756	0.016	0.104	0.918
Education, higher	0.023	−7.033	6.980	−0.152	−1.008	0.319	0.020	−6.633	7.025	−0.143	−0.944	0.350
Working	0.007	−3.767	7.038	−081	−0.535	0.595	0.008	−4.067	7.071	−0.087	−0.575	0.568
Weekly exercises	0.008	4.499	7.851	0.090	0.573	0.570	0.016	−1.399	1.679	−0.126	−0.833	0.409
**Sleep Measures**												
AHI (≥15) ^1^	0.011	−1.175	1.675	−0.106	−0.702	0.487	0.003	2.924	7.953	0.058	0.368	0.715
ISI (≥15) ^2^	0.006	−3.776	7.167	−0.080	−0.527	0.601	0.024	−7.316	7.141	−0.154	−1.024	0.311
ESS (>10) ^3^	0.009	4.223	6.709	0.096	0.629	0.532	0.002	2.176	6.766	0.49	0.322	0.749
**Mental Health (DASS-21 ^4^)**												
Depression (≥10)	0.031	−9.492	8.228	−0.175	−1.154	0.255	0.059	−13.175	8.132	−0.242	−1.620	0.113
Anxiety (≥8)	0.007	−5.333	9.789	−0.084	−0.545	0.589	0.022	−9.368	9.446	−0.147	−0.961	0.342
Stress (≥15)	0.006	4.337	8.331	0.080	0.521	0.605	0.000	0.095	8.382	0.002	0.011	0.991

^1^ Apnea–hypopnea index, ^2^ Insomnia severity index, ^3^ Epworth sleepiness scale, ^4^ The Depression, Anxiety and Stress Scale—21 Items.

**Table 3:** The average compliance rate for the morning and evening diaries on weekends and weekdays.

	Weekday Compliance (%) (*n* = 2892)	Weekend Compliance (%) (*n* = 1158)	*p*-Value
**Morning diary**	64.4	66.3	0.25
**Evening diary**	56.8	55.7	0.52

## Data Availability

The data analyzed for the purpose of this paper are not publicly available due to privacy and ethical restrictions.
